# Supplementary material for: Quantitative reconstruction of time-varying 3D cell forces with traction force optical coherence microscopy
Source: Sci Rep. 2019 Mar 11;9:4086. doi: 10.1038/s41598-019-40608-4 (PMC6411852; doi:10.1038/s41598-019-40608-4)
Supplement: Supplementary file 1 — Supplementary Information [file 41598_2019_40608_MOESM1_ESM.pdf]

Supplementary Information for:

Quantitative reconstruction of time-varying 3D cell forces with traction force optical coherence microscopy

Jeffrey A. Mulligan[1], Xinzeng Feng[2], Steven G. Adie[3,*]

[1] School of Electrical and Computer Engineering, Cornell University, Ithaca, New York 14853
[2] Institute for Computational Engineering and Sciences, The University of Texas at Austin, Austin, Texas 78712
[3] Nancy E. and Peter C. Meinig School of Biomedical Engineering, Cornell University, Ithaca, New York 14853

* Corresponding author: sga42@cornell.edu

Supplementary Methods

Computational image formation module

The procedures below detail the implementation of our computational image formation module (see Fig. 1). All image signal functions (denoted with an 'S') are complex valued. For each time point in a given time-lapse experiment, we will refer to the time using an integer designation. That is, t_i will refer to the time of the ith time point in an experiment with five-minute temporal sampling. For example, t_0 refers to zero minutes, t_1 refers to five minutes, and so on. All equations were designed for processing OCM data acquired with a Gaussian beam in a double-pass imaging configuration. All procedures were automated, except where indicated otherwise.

Initial volume reconstruction

Initial volumetric OCM images were reconstructed from raw spectral data via the standard operations of background subtraction, spectrum resampling, dispersion compensation, and the Fourier transform. The function $S_{\text{init}}(x,y,z,t_i)$ will refer to the image signal produced by this initial reconstruction. The full axial (z) range spanned by S_{init} contained two regions of interest, which were identified manually. The first was a 50 voxel thick region centered on the coverslip surface of the glass-bottomed petri dish. This set of depths will be referred to as $Z_{\text{coverslip}}$. The second was a 400 voxel (500 μm) thick region centered on the imaged cell (and consequently, the focal plane, which was aligned to the cell as described in the Methods). This set of depths will be referred to as Z_{cell}.

Coherence gate curvature removal

Due to the presence of non-idealities in our optical setup and/or sample stage setup, the coverslip surface appeared neither flat nor level in S_{init}. It was assumed that all deviations of the coverslip surface from flat and level were due to the presence of coherence gate curvature[1], and were therefore not representative of the 'true' sample structure. (The reasoning behind this assumption is detailed in the Supplementary Discussion.) To remove this coherence gate curvature, first, the axial position of the coverslip surface was estimated at each transverse position as

$$\hat{z}_c(x,y,t_i) = \underset{z\in Z_{\text{coverslip}}}{\arg\max} \left| S_{\text{init}}(x,y,z,t_i) \right| \tag{S.1}$$

where $\hat{z}_c(x,y,t_i)$ denotes the estimated axial depth of the coverslip surface. Next, linear least squares curve-fitting was performed at each time point to approximate $\hat{z}_c(x,y,t_i)$ with a 2D quadratic function in (x,y), given by

$$z_c(x,y,t_i) = a_{xx}(t_i)x^2 + a_{xy}(t_i)xy + a_{yy}(t_i)y^2 + a_x(t_i)x + a_y(t_i)y + a_0(t_i)\,. \tag{S.2}$$

Finally, the coherence gate curvature was removed via application of the Fourier shift theorem along the z-axis. The image signal produced by this procedure, $S_c(x,y,z,t_i)$, is defined as

$$S_c(x,y,z,t_i) = \mathrm{F}_z^{-1}\left[\mathrm{F}_z\left[S_{\text{init}}(x,y,z,t_i) \right] \exp\left(jk_z\left(\left(z_c(x,y,t_0) - a_0(t_0) \right) + \left(a_0(t_i) - a_0(t_0) \right) \right) \right) \right] \tag{S.3}$$

where F_z and F_z^{-1}, denote the forward and inverse 1D Fourier transform operations along z, respectively, k_z denotes axial spatial frequency (the Fourier conjugate variable to z), and $j = \sqrt{-1}$. Note that by using $z_c(x,y,t_0)$ for each time point instead of $z_c(x,y,t_i)$ in the expression above, we have assumed that all images in a time series exhibit the same degree of tilt and curvature, which would be expected if the optical system is not disturbed or altered during imaging. Consequently, all images in a time series are subjected to exactly the same degree of tilt/curvature removal. In this way, even if errors were present in the calibration stage of this procedure, all images in the time series received a *consistent* correction.

Upon completion, the coverslip surface was both flat and level, as desired, and located at the depth $z = a_0(t_0)$ at every time point. Before proceeding to further stages of image formation, the image signal phase at the (now flat and level) coverslip was extracted and saved as the function ϕ_c via the relation

$$\phi_c(x,y,t_i) = \angle S_c(x,y,a_0(t_0),t_i)\,. \tag{S.4}$$

This function was used later on during phase registration.

Focal plane registration

Again, due to the presence of non-idealities in our optical set up and/or sample stage setup, the focal plane appeared neither flat nor level in S_{init}. This focal plane curvature was not equivalent to the coherence gate curvature of the system. Therefore, the focal plane still exhibited a degree of curvature/tilt in the signal S_c. As a flat and level focal plane is ideal for standard, direct implementations of CAO, we devised a focal plane registration procedure for this study. The impact of this procedure is shown in Fig. 3 of the main text. Further discussion about this procedure may be found in the Results section of the main text, and the Supplementary Discussion.

Focal plane registration was performed in a manner similar to coherence gate curvature removal. First, the axial position of the focal plane was estimated from $S_c(x, y, z, t_0)$ to yield an estimate function $\hat{z}_f(x, y)$. This was achieved by leveraging the fact that the OCM signal magnitude (within the region Z_{cell}) was strongest at the focal plane. (For a given transverse position (x, y), local transverse averaging of the OCM signal magnitude was used to generate a depth-dependent intensity profile. $\hat{z}_f(x, y)$ was measured as the depth corresponding to the peak of this profile.) Next, linear least squares curve-fitting was performed to approximate $\hat{z}_f(x, y)$ with the function

$$z_f(x, y) = b_{xx}x^2 + b_{xy}xy + b_{yy}y^2 + b_x x + b_y y + b_0 \ . \tag{S.5}$$

Finally, the Fourier shift theorem was applied (along the *z*-axis) to yield, $S_f(x, y, z, t_i)$, defined here as

$$S_f(x, y, z, t_i) = \mathrm{F}_z^{-1}\left[\mathrm{F}_z\left[S_c(x, y, z, t_i)\right]\exp\left(jk_z\left(z_f(x, y) - b_0\right)\right)\right]. \tag{S.6}$$

Similar to the coherence gate curvature removal procedure, this operation applies a constant amount of tilt/curvature correction to all datasets in a time series. In the corrected image signal S_f, the (now flat) focal plane was located *near* the depth $z = b_0$ throughout the time-lapse dataset. The focal plane did not stay at a fixed depth due to the presence of small axial shifts of the sample stage throughout a given time-lapse experiment. However, due to the formulation of Eqn. (S.3), the axial position of the sample image remains fixed across all time points, even if the position of the focal plane drifted up and down relative to the sample. Only those depths in the range $z \in Z_{\text{cell}}$ needed to be retained after this step for further processing. Note that this operation deforms the image coordinate system away from of the (assumed) correct coordinate system established by the coherence gate curvature removal procedure. This effect was mitigated after the bead tracking step, during the calculation of bead displacements.

Phase registration

As shown in the Results section of the main text, phase registration was critical to the accurate reconstruction of volumetric images using CAO. Phase registration was achieved via a simple multiplication operation

$$S_{pr}(x, y, z, t_i) = S_f(x, y, z, t_i)\exp\left(-j\phi_c(x, y, t_i)\right), \tag{S.7}$$

where S_{pr} denotes the phase-registered image signal, and ϕ_c is defined as in Eqn. (S.4).

Bulk demodulation

Bulk demodulation was another critical step to achieve accurate reconstruction of volumetric images, given our implementation of CAO. Due to misalignments or tilts in the optical and/or sample stage setups, the image signal S_{pr} was not centered in the spatial frequency domain. Specifically, the signal was offset along the transverse spatial frequencies, k_x and k_y. First, this offset was measured via the operation

$$\left(k_{x,0}(t_i), k_{y,0}(t_i)\right) = \underset{(k_x, k_y)}{\arg\max}\left(\int\left|\tilde{S}_{pr}(k_x, k_y, k_z, t_i)\right|\mathrm{d}k_z\right), \tag{S.8}$$

where $\tilde{S}_{pr}(k_x, k_y, k_z, t_i)$ denotes the (spatial) 3D Fourier transform of $S_{pr}(x, y, z, t_i)$, and $\left(k_{x,0}(t_i), k_{y,0}(t_i)\right)$ denote the transverse spatial frequency offsets throughout the time-lapse experiment. In our experiments, these coefficients did not vary significantly over time, but we chose to allow our algorithm to be responsive to variations regardless, as the previous phase registration step is capable of generating image signals (S_{pr}) that exhibit variations. Note that if the image signal has a strong background signal (e.g., due to poor background subtraction), Eqn. (S.8) may not produce the correct result. Multiple methods can mitigate this problem, such as applying a median filter prior to evaluating the $\arg\max$ operator, or performing curve-fitting and peak-finding (such as with a quadratic or Gaussian profile) as a replacement for the $\arg\max$ operation. The spatial frequency offsets were removed via a demodulation operation

$$S(x, y, z, t_i) = S_{pr}(x, y, z, t_i)\exp\left(-j\phi_b(x, y, t_i)\right), \tag{S.9}$$

where $\phi_b(x, y, t_i) = k_{x,0}(t_i)x + k_{y,0}(t_i)y$.

Computational adaptive optics

Computational adaptive optics can be used mitigate the effects of both defocus and optical aberrations in volumetric OCT/OCM images[2]. In this study, no significant aberrations were present. Therefore, a direct (i.e., non-iterative) implementation of CAO was used to mitigate only the effects of defocus throughout the 500×500×500 μm^3 volume containing the imaged cell. First, the axial position of the focal plane z_{focus} was found for each time point by finding the depth $z \in Z_{\text{cell}}$ with the strongest average signal magnitude. Then, defocus was removed from the imaged volumes via the operation

$$S_{\text{refocus}}(x, y, z, t_i) = \mathrm{F}_{x,y}^{-1}\left[\mathrm{F}_{x,y}\left[S(x, y, z, t_i)\right]\exp\left(2j(z - z_{\text{focus}}(t_i))\sqrt{(nk_c)^2 - k_x^2 - k_y^2}\right)\right], \tag{S.10}$$

where $\mathrm{F}_{x,y}$ and $\mathrm{F}_{x,y}^{-1}$ denote the forward and inverse 2D Fourier transform operations along the transverse (*x*,*y*) dimensions, respectively, k_c denotes the central wavenumber of the system ($2\pi / 800$ nm^{-1} for our system), and n denotes the refractive index of the substrate material (for Matrigel, a value of n=1.34 was used). This operation resulted in volumetric images which exhibited a transverse resolution of ~2 μm throughout the axial range spanned by Z_{cell}.

Accounting for 'full FOV' and 'reduced FOV' images

As described in the Methods, two types of images were used in our TF-OCM procedure. For each time point t_i, nine separate images were acquired: one 'full FOV' image (spanning a 500×500 μm^2 transverse FOV) and eight 'reduced FOV' images (spanning a 125×125 μm^2 transverse FOV). The equations in the sections above did not account for the fact that each time point t_i has several associated images. This detail was omitted from the previous sections to allow for easier comprehension of the concepts and processes underlying our computational image formation module. In order to apply our module to our multi-acquisition scheme, the following modifications were applied to the procedures detailed above:

1. The calibration steps for coherence gate curvature removal, focal plane registration, bulk demodulation, and computational adaptive optics (i.e., the measurements of $\hat{z}_c(x, y, t_i)$, $\hat{z}_f(x, y)$, $\left(k_{x,0}(t_i), k_{y,0}(t_i)\right)$, and $z_{\text{focus}}(t_i)$, respectively) were performed using *only* the 'full FOV' image associated with each time point t_i. This assumes that these functions should remain constant for all images acquired at a given time point. This follows from the fact that these functions are determined by the structure of the entire sample and optical setup, which should vary slowly in time.
2. The calibration step for phase registration (i.e., the measurement of $\phi_c(x, y, t_i)$) was performed for each image independently. That is, it was assumed that the phase fluctuations encoded in $\phi_c(x, y, t_i)$ could change every single time a new image was acquired (even when images correspond to the same time point). This follows from the fact that the phase of OCM images is highly sensitive to vibrations, laser fluctuations, etc. and therefore may fluctuate rapidly in a non-repeating fashion.

Traction force microscopy module

Pre-processing of bead position data

Time-varying bead positions were measured and tracked from OCM image data as described in the Methods. Define M as the total number of tracked beads. The position of each bead $m \in \{1,...,M\}$ at time t_i is given by a column vector:

$$\mathbf{r}_{\mathrm{raw},m}(t_i) = \left\langle x_{\mathrm{raw},m}(t_i), y_{\mathrm{raw},m}(t_i), z_{\mathrm{raw},m}(t_i) \right\rangle \tag{S.11}$$

Note that this position data is defined in the warped coordinate system, established by the focal plane registration procedure in Eqn. (S.6). Bead positions in the 'correct' coordinate system (established by the coherence gate curvature removal procedure, Eqn. (S.3)) were therefore obtained via

$$\mathbf{r}_m(t_i) = \mathbf{r}_{\mathrm{raw},m}(t_i) + \left\langle 0, 0, z_f\left(x_{\mathrm{raw},m}(t_i), y_{\mathrm{raw},m}(t_i)\right) - b_0 \right\rangle, \tag{S.12}$$

where $z_f(x,y)$ and b_0 are as defined in Eqn. (S.5).

Motion correction for bead displacement data

The position data obtained from Eqn. (S.12) contained residual bulk motion artifacts. Zero-order artifacts corresponded to a translational shift of the bead positions between time points, and likely originated from drift of the sample or sample stage. First-order artifacts corresponded to small linear distortions of the entire volume spanned by the bead position data. Such artifacts could have resulted from algorithm assumption failures (see the Supplementary Discussion), small calibration errors in our automated computational image formation procedures, and/or from swelling, shearing, and/or small rotational drift of the hydrogel medium (e.g., due to non-uniform temperatures). Motion-corrected bead data $\mathbf{r}_{\mathrm{corr},m}(t_i)$ were computed via the equation

$$\mathbf{r}_{\mathrm{corr},m}(t_i) = \mathbf{r}_m(t_i) - \mathbf{u_0}(t_i) - \mathbf{A}(t_i)\mathbf{r}_m(t_i), \tag{S.13}$$

where $\mathbf{u_0}(t_i)$ is a time-varying vector which describes zero-order motion artifacts, and $\mathbf{A}(t_i)$ is a time-varying 3×3 tensor which describes first-order motion artifacts. Both motion correction parameters, $\mathbf{u_0}(t_i)$ and $\mathbf{A}(t_i)$, were estimated from the bead position data $\mathbf{r}_m(t_i)$. Specifically, both $\mathbf{u_0}(t_i)$ and $\mathbf{A}(t_i)$ were computed simultaneously via a linear least squares fit, achieved by solving the system of equations

$$\mathbf{R}(t_i) - \mathbf{R}(t_{\max}) = \mathbf{R}_{\mathrm{V}}(t_i)\left[\mathbf{u_0}(t_i) \quad \mathbf{A}(t_i)\right]^{\mathrm{T}} \tag{S.14}$$

where $\mathbf{R}(t_i)$ denotes a M×3 matrix of concatenated bead position data, given by

$$\mathbf{R}(t_i) = \begin{bmatrix} \mathbf{r}_1(t_i)^{\mathrm{T}} \\ \vdots \\ \mathbf{r}_M(t_i)^{\mathrm{T}} \end{bmatrix}, \tag{S.15}$$

and $\mathbf{R}_{\mathrm{V}}(t_i)$ is a Vandermonde matrix given by

$$\mathbf{R}_{\mathrm{V}}(t_i) = \left[\mathbf{1}^{M\times 1} \quad \mathbf{R}(t_i)\right], \tag{S.16}$$

where $\mathbf{1}^{M\times 1}$ denotes a M×1 column vector with every entry equal to 1. Note that $t_{\max} = t_{36}$ = 3 hours corresponds to the last time point in our time-lapse experiment protocol, which we used to define the reference position of each bead.

Equation (S.14) relies on the assumption that the effects of CTF-induced substrate deformations were negligible across a large fraction of the beads tracked in the 500×500×500 μm^3 FOV of our images, such that the output parameters of the fitting operations were not severely affected. (Further details are provided in the Supplementary Discussion.) Bead displacement data was finally computed as

$$\mathbf{u}_m(\mathbf{r}_{\text{corr},m}(t_{\max}), t_i) = \mathbf{r}_{\text{corr},m}(t_i) - \mathbf{r}_{\text{corr},m}(t_{\max}) \ . \tag{S.17}$$

This data was used as input for the CTF reconstruction procedure described in the Methods section.

Pre-processing and motion correction for cell surface mesh data

In order to ensure that the bead displacement data (Eqn. (S.17)) and cell surface mesh data (acquired as described in the Methods) are defined in the same coordinate systems, all position corrections applied to the bead position data by Eqns. (S.12) and (S.13) must also be applied to each node of the cell surface mesh data. Let Q denote the total number of nodes in the cell surface mesh at time t_i. For each node $q \in \{1,...,Q\}$, with raw position denoted by the column vector $\mathbf{r}_{\text{raw},q}(t_i)$, the effects of focal plane registration were removed, as in Eqn. (S.12), to obtain

$$\mathbf{r}_q(t_i) = \mathbf{r}_{\text{raw},q}(t_i) + \left\langle 0, 0, z_f\left(x_{\text{raw},q}(t_i), y_{\text{raw},q}(t_i)\right) - b_0 \right\rangle . \tag{S.18}$$

This was followed by motion correction, as in Eqn. (S.13), to obtain the corrected node positions

$$\mathbf{r}_{\text{corr},q}(t_i) = \mathbf{r}_q(t_i) - \mathbf{u}_{\mathbf{0}}(t_i) - \mathbf{A}(t_i)\mathbf{r}_q(t_i) \ , \tag{S.19}$$

where $\mathbf{u}_{\mathbf{0}}(t_i)$ and $\mathbf{A}(t_i)$ use the same values/coefficients as those obtained from Eqn. (S.14).

Methods for figure generation

Impact of focal plane registration (Figs. 3(b) and 3(c))

The panels for Figs. 3(b) and 3(c) were generated using the procedure below.

Two different volumetric images were generated from the same raw image data, using our computational image formation procedure. The volumetric image used to produce Fig. 3(b) was generated without using focal plane registration, whereas the volumetric image used to produce Fig. 3(c) was generated with focal plane registration. To assess the impact of focal plane registration, both volumetric images were analyzed using the same procedure:

1. A 50 μm thick region, centered 50 μm above the focal plane, was extracted from the full volumetric image.
2. A 2D image was generated via a maximum intensity projection of this sub-volume, taken along the *z*-axis.
3. This 2D image was subjected to single-level thresholding, with the threshold set as the 99th percentile of the image histogram. This resulted in a binary image, where regions with a value of '1' corresponded to regions with strong scattering signals in the original image.
4. The centroid of each 'object' (i.e., a region of connected pixels with a value of '1') in the binary image was computed and recorded. Each object was assumed to represent at least one scattering bead.
5. For each centroid computed in step 4, the following was performed:
    a. Extract an 11 pixel × 11 pixel region (centered on the centroid) within the image from step 2.
    b. Up-sample the extracted region by a factor of 100 (using MATLAB's *imresize* function)
    c. Threshold this up-sampled region at one-half its maximum value, to generate a binary image.
    d. Compute the area and perimeter (*A* and *P*) of the largest object in this binary image.
    e. Compute the 'circularity' $C = P^2/(4\pi A)$. If $C < 2$, the object is assumed to be approximately circular, and therefore corresponds to a single bead, whose full-width-at-half-maximum (FWHM) diameter is taken to be $D = 2\sqrt{A/\pi}$. Otherwise, the object is discarded and ignored (as it may contain overlapping bead images or have an insufficient signal-to-noise ratio).
6. Each 11×11 region from step 5 which was determined to contain a single bead was assigned a color value (see the color bar in Fig. 3) based on the FWHM bead diameter *D* computed for that region. This region was inserted at its appropriate location in a synthetic image, corresponding to the images in Figs. 3(b) and 3(c).

Average displacement with respect to distance from the cell centroid (Fig. 5, right panels)

'Average deformation magnitude' (shown in the right panels of Fig. 5) was computed using the procedure below. All mesh/position/deformation data refer exclusively to the data of a given cell (1, 2, or 3) and time point (in the range $t_i = t_0,\ldots,t_{\max}$).

1. Compute the centroid of each element of the cell surface mesh.
2. Compute the centroid of the cell body as the mean position of these element centroids.
3. Compute the distance of each bead to the cell centroid.
4. For each distance *d* depicted in the right panels of Fig. 5
    a. Identify all beads whose position is within the range d±20 μm from the cell centroid.
    b. Compute the mean of the magnitude of the deformation vectors for this set of beads.

Measurement of total force (Figs. 8 and 9)

The time-varying total force (depicted in Figs. 8 and 9) was computed as described below. All mesh/position/traction data refer exclusively to the data of a given cell (1-10) and time point (in the range $t_i = t_0,\ldots,t_{\max}$).

As described in the Methods, the reconstructed CTF distribution must satisfy force and moment balance (as is required by the quasi-static/non-accelerating state of the cell). Therefore, calculation of the 'total force' exerted by the cell cannot be performed via a summation of the traction forces exerted by each element in the cell surface mesh (as the result would trivially be zero for any cell at any time). Instead, we measured the 'total force' by 1) measuring the direction along which cell forces were maximally oriented, followed by 2) 'slicing' the cell with a plane perpendicular to this direction, such that the force exerted by each 'half' of the cell (in the direction normal to the plane) was maximal, and 3) taking this maximal force to be the 'total force'. The equations below give precise definitions for this process.

Let $\hat{\mathbf{n}}$ be a 3D normal vector (in column format) whose direction is described by a pair of angular coordinates (θ, φ) such that its components in *x*, *y*, and *z* are given by

$$\hat{\mathbf{n}}(\theta,\varphi) = \langle \cos\theta\sin\varphi, \sin\theta\sin\varphi, \cos\varphi \rangle \ . \tag{S.20}$$

Let the principal axis of stress be defined as a normal vector which is maximally aligned with the stress distribution exerted by the cell. That is, let the principal axis of stress be defined as $\hat{\mathbf{n}}_p\left(\theta_p, \varphi_p\right)$, where the principal angles $\left(\theta_p, \varphi_p\right)$ are computed as

$$\left(\theta_p, \varphi_p\right) = \underset{(\theta,\varphi)}{\arg\max} \sum_{l=1}^{N} \left| \left(a_l \mathbf{t}_l\right) \bullet \hat{\mathbf{n}}(\theta,\varphi) \right| \tag{S.21}$$

where N is the number of elements in the cell surface mesh, a_l and $\mathbf{t}_l$ denote the area and reconstructed traction *stress* of element l of the cell surface mesh, respectively, and '$\bullet$' denotes the vector dot product operator. Once the principal axis of stress is identified, the cell can be 'sliced' perpendicular to the principal axis with a plane

$$\left(\langle x, y, z\rangle - \beta\hat{\mathbf{n}}_p\right) \bullet \hat{\mathbf{n}}_p = 0 \ , \tag{S.22}$$

where β denotes the 'offset position' of the plane with respect to the origin. To determine whether or not a given element of the cell surface mesh is on one side or the other of this plane, we define an indicator function

$$\chi_\beta\left(\mathbf{c}_l, \hat{\mathbf{n}}_p\right) = H\left(\left(\mathbf{c}_l - \beta\hat{\mathbf{n}}_p\right) \bullet \hat{\mathbf{n}}_p\right) \tag{S.23}$$

where $\mathbf{c}_l$ denotes the centroid of element l of the cell surface mesh, and H denotes the Heaviside step function. The total force exerted by the cell was then approximated as

$$F_{\text{total}} = \max_\beta \left| \sum_{l=1}^{N} \left(\left(a_l \mathbf{t}_l\right) \bullet \hat{\mathbf{n}}_p\right) \chi_\beta\left(\mathbf{c}_l, \hat{\mathbf{n}}_p\right) \right| \ . \tag{S.24}$$

The maximization over β finds the location of the 'slicing' plane which yields the largest measurement of total force. Note that Eqn. (S.24) performs a summation of the CTF components parallel to the principal axis of stress.

Supplementary Discussion

Phase registration, bulk demodulation, and accurate analysis of volumetric images reconstructed with CAO

As stated in the main text, the phase registration and bulk demodulation procedures used in this study mitigate distortions that can appear in volumetric images reconstructed with CAO. The optimal implementations of these methods (and the consequences of not using them) may vary between optical systems, experimental setups, and/or image reconstruction algorithms (such as varying implementations of CAO and related methods [2-4]). Here, we discuss the origin of the image distortions shown in Fig. 2 and Supplementary Movie 1, so that our findings and algorithms may be more readily adapted to other settings.

Bulk modulations of the OCM signal can result from a tilt/misalignment in optical components or the sample stage. Phase instabilities, on the other hand, may originate from a number of sources, including vibrations, power fluctuations, temperature changes, etc. In beam-scanning systems, temporal fluctuations of the signal phase between A-scans result in transverse spatial fluctuations in the phase of the volumetric image signal[5,6]. This effect will be most pronounced along the 'slow' scanning axis, in particular. If the signal phase varies significantly over length scales similar to the transverse PSF width at a particular depth in the original image, CAO and related algorithms may not be able to restore focal plane resolution in post-processing.

As can be seen from the results in Fig. 2 and Supplementary Movie 1, our system exhibited sufficient phase stability, such that rapid phase fluctuations did not hinder the refocusing performed by our CAO algorithm. However, obtaining a high-resolution volumetric image was not sufficient for TF-OCM. This is because slowly varying phase fluctuations, spanning tens to hundreds of micrometers, caused image distortions over long length scales while having little apparent impact on the final transverse resolution. (The math underlying this principle is outlined in the paragraphs below.) Without proper corrections, these distortions would have hindered accurate quantitative analysis of the sample structure (e.g., the measurement of bead displacements induced by CTFs). Therefore, it can be understood that the phase stability requirements for performing accurate quantitative image analysis essential to TF-OCM are stricter than the requirements for only obtaining high-resolution images.

To better understand the origin of the distortions discussed above, we can examine what would happen if we applied our CAO procedure *without* first performing phase registration and bulk demodulation (this corresponds to the green channel of Fig. 2 and Supplementary Movie 1). We will restrict ourselves to a single time point in a time-lapse dataset, so the dependence on time t_i will be dropped, for simplicity. We start with our uncorrected and defocused signal, which corresponds to S_f, defined in Eqn. (S.6). We can relate this signal to the phase registered and bulk-demodulated signal S (Eqn. (S.9)) via the relation

$$S_f(x,y,z) = S(x,y,z)\exp\left(j(k_{x,0}x + k_{y,0}y)\right)\exp\left(j\phi_c(x,y)\right). \tag{S.25}$$

We assume for now that ϕ_c is very small/negligible. That is, we will first ignore the effects of phase instability and only look at the effects of neglecting the bulk demodulation procedure. We will discuss the impact of neglecting to perform phase registration at the end. Using our assumption, we can approximate the 2D Fourier transform of Eqn. (S.25) along the transverse dimensions as

$$\tilde{S}_f(k_x,k_y,z) \approx \tilde{S}(k_x - k_{x,0}, k_y - k_{y,0}, z). \tag{S.26}$$

That is, the Fourier transform of S_f is shifted in the spatial frequency domain relative to the corrected image signal $\tilde{S}(k_x,k_y,z)$, as defined by the pair $(k_{x,0},k_{y,0})$. As described previously, we perform CAO on S via the operation defined in Eqn. (S.10). However, if we instead substitute S_f into Eqn. (S.10), using the assumptions above, we obtain a corrupted image signal whose 2D transverse Fourier transform is given by the expression

$$\tilde{S}_{\text{artif}}(k_x,k_y,z) = \tilde{S}(k_x - k_{x,0}, k_y - k_{y,0}, z)\exp\left(2j(z - z_{\text{focus}})\sqrt{(nk_c)^2 - k_x^2 - k_y^2}\right), \tag{S.27}$$

where $\tilde{S}_{\text{artif}}(k_x, k_y, z)$ is our computationally refocused data, with distortion artifacts present. We can arrive at an approximate space domain representation of this expression by using small-angle approximations. Specifically, if we assume that $k_{x,0}, k_{y,0} \ll nk_c$, and that $k_x, k_y \ll nk_c$ for all (k_x, k_y) spanned by the signal $\tilde{S}(k_x, k_y, z)$, then the expressions $\sqrt{(nk_c)^2 - k_x^2 - k_y^2}$ and $\sqrt{(nk_c)^2 - (k_x - k_{x,0})^2 - (k_y - k_{y,0})^2}$ may approximated by their first-order Taylor series. Using this approximation, we obtain the distorted image signal $S_{\text{artif}}(x, y, z)$ given by

$$S_{\text{artif}}(x,y,z) = S_{\text{refocus}}(x',y',z)\exp\left(j\left(k_{x,0}x' + k_{y,0}y'\right)\right)\exp\left(j\left(z - z_{\text{focus}}\right)\left(k_{x,0}^2 + k_{y,0}^2\right)\Big/nk_c\right) \quad \text{(S.28a)}$$

$$x' = x - 2(z - z_{\text{focus}})k_{x,0}\big/nk_c \quad \text{(S.28b)}$$

$$y' = y - 2(z - z_{\text{focus}})k_{y,0}\big/nk_c \quad \text{(S.28c)}$$

where S_{refocus} is defined as in Eqn. (S.10). The magnitude/intensity profile of S_{artif} is equivalent to that of the desired signal S_{refocus}, with the addition of a depth-dependent 2D translation artifact, given by Eqns. (S.28b) and (S.28c). This translation increases with distance from the focal plane and has opposite signs on opposite sides of the focal plane. This translation artifact is therefore consistent with that depicted in Fig. 2.

If our initial assumption that ϕ_c is negligible fails (i.e., that the effects of phase instability are not negligible), the analysis becomes more complicated. However, if we choose to examine a sufficiently small transverse region of our image signal, we may approximate ϕ_c with a local first-order Taylor series. Performing an analysis analogous to the one above, we obtain a more complete version of coordinate transformation described by Eqns. (S.28b) and (S.28c):

$$x' = x - 2\left(z - z_{\text{focus}}\right)\left(k_{x,0} + \tfrac{\partial}{\partial x}\phi_c(x,y)\right)\Big/nk_c \quad \text{(S.29a)}$$

$$y' = y - 2\left(z - z_{\text{focus}}\right)\left(k_{y,0} + \tfrac{\partial}{\partial y}\phi_c(x,y)\right)\Big/nk_c \quad \text{(S.29b)}$$

These translations will exhibit the same properties as those from Eqns. (S.28b) and (S.28c), but can vary as a function of transverse position. In addition, as we expect the gradient of ϕ_c to be largest along the slow scanning axis, we would expect the translations encoded by these coordinate transformations to be largest along the slow axis. These behaviors are consistent with translation/motion artifacts shown in Supplementary Movie 1.

The integration of CAO with applications requiring accurate quantitative analysis of sample structure requires a means to maintain or synthesize phase stability across the full image volume. Meeting this requirement is essential even if computational methods are used only to mitigate defocus (as was done in this study). If CAO is also used to correct more general wavefront distortions (e.g., astigmatism and higher order aberrations), the consequences of neglecting phase registration and bulk demodulation will be more complex and perhaps more severe than the effects described above[7]. An example of this phenomenon may be found in Fig. 3 of Supplementary Ref. [7].

Assumptions and failure modes of coordinate transformations in TF-OCM

In general, we recommend that any OCM system used to perform TF-OCM be aligned so as to minimize coherence gate curvature, focal plane curvature, and any other non-idealities as much as is feasible. This will minimize the role that is played by the procedures discussed below, and by extension will minimize the impact of any erroneous calibrations or assumption failures that emerge during processing. If the computational procedures below must still be employed, we recommend taking this discussion into consideration during both software design and data interpretation.

Coherence gate curvature removal

Our coherence gate curvature removal procedure assumes that the ‘true’ structure of the coverslip surface of our samples ought to appear flat and level in a given volumetric image. Although the first assumption is reasonable, the latter assumption may fail to account for any true physical tilt of the sample with respect to the optical axis. The appearance of sample tilt in an uncorrected OCT/OCM image could also result from variations in the scanning beam’s optical path length across the FOV due to misalignments or tilts elsewhere in the optical system. This makes any measurement of the ‘true’ degree of sample tilt from an image ambiguous and difficult to assess. In addition, because

the coherence gate curvature removal algorithm flattens and levels the sample via a transversely-varying vertical shearing transformation (i.e., each A-scan is vertically shifted until the coverslip appears flat and level), it does not perform the 3D rotation necessary to truly remove sample tilt. Such an operation would require significant additional computation (in the form of resampling) and could introduce errors/noise to the phase data that the latter stages of phase registration and CAO rely upon. Given this information, we maintained the assumption of a level surface for its computational convenience and for consistency of the processing workflow across multiple experiments.

It should also be noted that coherence gate curvature removal may be performed through alternative methods[1]. For example, the procedure in Supplementary Ref. [1] performs all the same operations as our dispersion compensation, coherence gate curvature removal, and phase registration algorithms simultaneously. However, we chose to split up this functionality across the multiple algorithms described in the Supplementary Methods, as this allowed for greater flexibility and computational convenience when processing the numerous large datasets used in this study.

Focal plane registration

Focal plane registration uses the same type of coordinate transformation as coherence gate curvature removal. As such, the shearing operations that it employs are only approximations of the true transformation required to obtain a flat/level focal plane and a corresponding transversely invariant PSF compatible with our CAO algorithm. As a result, even though focal plane registration only transforms the axial coordinate system (i.e., $z \rightarrow z' = f(x, y)$), a very large tilt or curvature in the raw focal plane may result in additional transverse coordinate transformations after the application of CAO (e.g., $x \rightarrow x' = f(x, y, z)$). We have not yet characterized what degree of focal plane tilt or curvature would produce significant image distortions, and have therefore relied on the assumption that our data has not been severely affected by any such artifacts. If this assumption was invalid, the images generated by our computational image formation module would be distorted, contributing to errors in our reconstructed CTFs. However, this would only impact TF-OCM results generated by the specific imaging system used in this study, and would not prevent other imaging systems from performing TF-OCM using our methods.

If focal plane tilt/curvature is present in an imaging system, and the effect of possible distortions is a concern, then the mechanisms of CTF reconstruction can tell us what optical configuration would have the minimum impact on the accuracy of CTF reconstructions. Since CTF-induced substrate deformations rapidly decay with distance from the cell body in a linear elastic medium, substrate deformation measurements close to the cell body will have the largest impact on the accuracy of CTF reconstructions[8]. Therefore, accurate image reconstruction is most essential in the immediate vicinity of the cell body. If the focal plane was locally flat and level near the cell body, then focal plane registration would not substantially alter the image data in this region. Therefore, the distortions discussed above would be minimal, and accurate CTF reconstruction would be feasible. By extension, if the focal plane was locally curved/tilted only in regions far away from the cell body, then any significant image distortions in these regions would have a lesser impact on CTF reconstruction accuracy than if they were present near the cell body.

Bulk demodulation and phase registration

The impact of coverslip phase registration and bulk demodulation on data processed with CAO have already been discussed at the beginning of the Supplementary Discussion. However, we would like to alert any readers interested in replicating our methods that it is critical that these two procedures are performed in the correct order. Coverslip phase registration must take place first. As we saw in our own data, the numerous local variations in ϕ_c caused the image data to become ‘smeared’ across the spatial frequency domain, especially along the slow scanning axis, where phase instabilities were most prominent. Once ϕ_c was removed by coverslip phase registration, the spatial frequency domain data took on a more typical Gaussian profile (as would be expected when imaging with a Gaussian beam). The bulk demodulation coefficients could then be measured by finding the peak of this restored Gaussian profile. However, even small errors in the localization of this peak could result in residual deformation artifacts in the image data. If localization errors differ across time points, this could create first-order bulk motion artifacts in the bead displacement data, which must then be removed with a motion correction procedure, such as the one described previously in the Supplementary Methods. As ϕ_c can vary dramatically between images, failing to perform phase registration prior to bulk demodulation could result in severe errors during the calibration stage of our bulk demodulation procedure.

Motion correction

The motion correction procedure (which was applied only to bead displacement data and cell surface mesh data, and *not* to OCM images) attempted to remove any residual motion artifacts that were not corrected by previous data

processing procedures. As described in the Supplementary Methods, the zero- and first-order terms used to perform this correction were determined by linear least squares fitting, using the assumption that CTF-induced bead displacements would have a negligible impact on the results of the fit. This method of fitting is vulnerable to outliers and does risk erroneously incorporating cell-induced displacements into the correction functions, $\mathbf{u_0}(t_i)$ and $\mathbf{A}(t_i)$. To better avoid this risk in the future, we recommend measuring deformations over a larger volumetric FOV, reducing the weighting of bead displacements measured near the cell body, and/or using alternative robust regression models (such as nonlinear metric functions, mixture models, etc.). In general, motion correction may be thought of as a type of high-pass filter (in the space-domain), applied to remove deformations spanning volumes too large to be caused by the cell. Any alternative filtering schemes should be optimized with this objective in mind. Note that low-pass filtering (to remove deformation fluctuations spanning volumes too small to be caused by the cell) was not directly performed in this study. Instead, low-pass filtering took place implicitly via the Tikhonov regularization used by our CTF reconstruction procedure[9].

Validity of linear elastic mechanical model

In order to reconstruct time-varying CTFs, our FEM solver employed a standard linear elastic model under the infinitesimal strain approximation. Any deviations of the Matrigel substrate's true behavior from this model could introduce errors to the final reconstructed CTFs. The linear elastic model can be a poor model for many biopolymers (such as collagen), which tend to exhibit nonlinear elastic and/or viscoelastic behaviors. Moreover, if cells induce very large deformations in the surrounding substrate, infinitesimal strain theory may no longer be sufficient to describe substrate deformations, requiring a generalization to finite strain theory. In order to assess the validity of the model used for this study, we tested our Matrigel substrate for both nonlinear and viscoelastic behaviors, and quantified discrepancies between the infinitesimal and finite strain tensors computed from our experimentally measured bead displacement data.

Bulk rheology testing for nonlinearity and viscoelasticity

Concerns regarding nonlinear and/or viscoelastic effects were raised during the peer review process for this study. At that time, our original batch of Matrigel had been exhausted, and a new batch was used for additional testing. Using the procedure outlined in the Methods section of the main text, the Young's modulus of the Matrigel substrates prepared from the new batch was found to be approximately 104 Pa. This is ~15% stiffer than the Young's modulus (90 Pa) measured from the original Matrigel batch used for our time-lapse experiments and CTF reconstructions. We have assumed that despite this difference, any tests performed with the new Matrigel batch should provide a reasonable approximation of the mechanical properties of the original batch. Three distinct samples were tested for nonlinear behavior via an oscillating (0.1 Hz) strain sweep test over a strain range of 2-20% (raw data is depicted in Fig. S.1). For each sample, the Young's modulus deviated from the mean for that sample by less than ±3%, suggesting that our Matrigel samples exhibit approximately linear behavior for strains up to at least 20%.

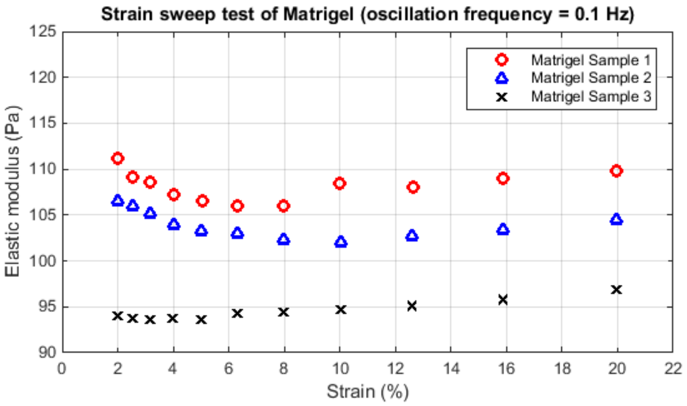

Figure S.1. Strain sweep test of Matrigel. The Matrigel samples exhibited approximately linear behavior, as indicated by an approximately constant stiffness over a strain range of 2-20%.

The same three samples used for the strain sweep test were also subjected to a stress relaxation test (under an applied strain of 2%) to test the potential impact of viscous effects. The stress relaxation curves (raw data depicted in Fig. S.2) were fit to the function $\sigma(t) = c_1 + c_2 e^{-t/\tau}$ via the MATLAB function *lsqcurvefit* to obtain the values of (c_1, c_2, τ). The output fitting parameters for each sample were (0.28 Pa, 0.56 Pa, 97.6 s), (0.18 Pa, 0.52 Pa, 122.5 s), and (0.24 Pa, 0.37 Pa, 117.5 s), respectively, with residual norms of 0.0157, 0.0424, and 0.0138, respectively. The stress relaxation constants τ are consistent with previously published characterizations of Matrigel[10], and do imply that Matrigel exhibits a degree of viscoelastic behavior. We can use the viscoelastic mechanical framework by Toyjanova *et al.*[11] to estimate the impact of neglecting viscous effects during CTF reconstruction. Given the applied strain (2%) of our stress relaxation test, the equilibrium shear modulus μ and non-equilibrium shear modulus μ_{neq} can be obtained by the relations $\mu = c_1 / 0.02$ and $\mu_{\text{neq}} = c_2 / 0.02$. The ratio μ_{neq} / μ then takes a mean value of approximately 2.14. Using this ratio, a mean relaxation time of $\bar{\tau} = 112.5$ seconds, and Fig. 6 of Supplementary Ref. [11], we estimate that applying a purely elastic model to analyze our samples will underestimate CTFs by <5%, compared to the viscoelastic FEM solver described in that study[11]. This suggests that, for this study, viscous effects may be reasonably neglected. Combined with the nonlinearity test above, our mechanical characterization data suggest that a linear elastic model is likely to provide sufficiently accurate CTF reconstructions for this study. However, it is important to note that the future application of TF-OCM in other, less ideal materials (such as collagen) may benefit from the incorporation of nonlinear and/or viscoelastic models to obtain more accurate CTF reconstructions.

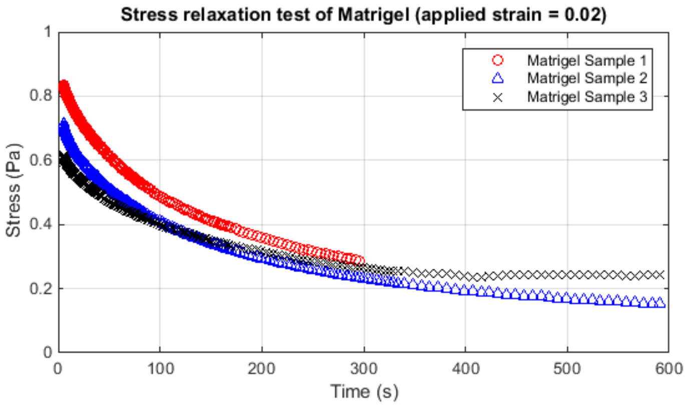

Figure S.2. Stress relaxation test of Matrigel. Parameters obtained from curve-fitting to these data were used to show that neglecting viscous effects is not expected to severely impact CTF reconstruction accuracy for this study.

Testing validity of the infinitesimal strain approximation

(Note that some of the variables/notation used in the equations below overload the variables/notation defined previously in this document. This has been done in order to maintain consistency between the equations below and Supplementary Ref. [12].)

1) Validation: Strain estimation using moving least squares method.

Consider a test scalar function $g(x, y, z) = \sin(x) + z^2 \sin(y)$ defined on a unit cube $[0,1]^3$. We use the moving least squares (MLS) method[12] to estimate the value of the function as well as its (spatial) derivatives, given a set of n point measurements of the function. Details can be found in Supplementary Ref. [12]. Briefly, here we adopt a cubic polynomial basis and the following exponentially decaying weight function:

$$f(\mathbf{x} - \mathbf{b}_i) = \begin{cases} \dfrac{\exp(1 - d^2 / d_m^2) - 1}{e - 1}, & d \le d_m \\ 0, & d > d_m \end{cases}, \quad d = |\mathbf{x} - \mathbf{b}_i| \tag{S.30}$$

where $\mathbf{x}$ is the point of interest, $\mathbf{b}_i$ is the position of the i-th measurement point $(1 \le i \le n)$, and d_m is a cut-off distance which we choose to be three times the mean separation distance of the measurement points. Different from Eqn. (7a) in Supplementary Ref. [12], we define

$$\mathbf{A} = r\mathbf{I} + \sum_{i=1}^{n} f(\mathbf{x}-\mathbf{b}_i)\mathbf{P}(\mathbf{b}_i)\mathbf{P}^T(\mathbf{b}_i), \tag{S.31}$$

where r is a regularization coefficient. In general, r reduces the condition number of the matrix $\mathbf{A}$ and improves the numerical stability of the MLS method. Figure S.3 shows a typical profile of the approximated value of the test function g and its derivatives at a random point in the unit cube as r varies. As r decreases, the MLS approximations (solid lines, black: g, red: $\partial g/\partial x$, green: $\partial g/\partial y$, blue: $\partial g/\partial z$) approach the true values (dotted lines) with less than 5% relative error. In the calculation, 50 measurements points were randomly sampled in the unit cube with a mean distance of approximately 0.177. Among them, the MLS weights of twenty measurement points are greater than 0.05 at the point of interest. We note that when the point of interest is close to the boundary of the unit cube, some estimated derivatives could yield significant numerical error (see Fig. S.4). For this reason, in the next section, we only applied the MLS method near the center of the imaging domain where the cell-induced displacement is maximal.

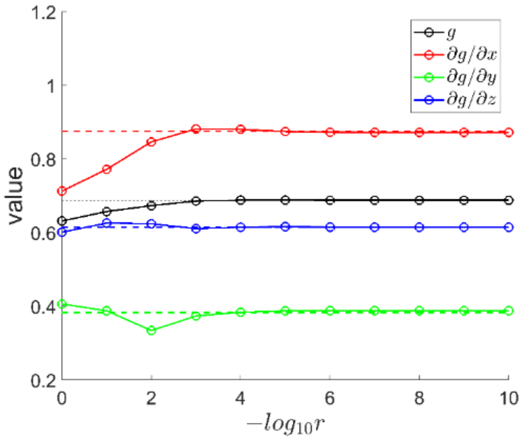

Figure S.3. MLS approximation of the test function (black) and its derivatives (red: x-derivative, green: y-derivative, blue: z-derivative) at the point of interest [0.5063, 0.4856, 0.6585] as the regularization coefficient r varies. As r decreases (from left to right), the MLS approximation approach the true values (dotted lines).

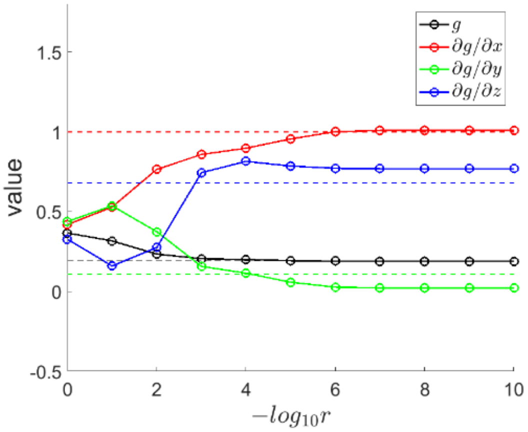

Figure S.4. MLS approximation of the test function (black) and its derivatives (red: x-derivative, green: y-derivative, blue: z-derivative) at the point of interest [0.0467, 0.9232, 0.4254] as the regularization coefficient r varies. As r decreases (from left to right), some approximated derivatives

have significant error from the true values (dotted lines), e.g., for the blue and green curves, due to the fact that the point of interest is near the boundary of the domain.

2) Results for experimental data

We use the above approach to validate that the cell-induced deformations in our experiment are approximately linear over time (i.e., that the infinitesimal strain tensor is a good approximation of the more general finite strain tensor). To this end, we estimate the displacement gradient $\nabla \mathbf{u}$ at multiple locations where the cell-induced bead displacement is maximal by applying the MLS method on each component of the displacement. This allows us to calculate the finite Green's deformation tensor $\mathbf{E} = \frac{1}{2}\left(\nabla \mathbf{u} + \nabla^T \mathbf{u} + \nabla^T \mathbf{u} \nabla \mathbf{u}\right)$ as well as the infinitesimal strain tensor $\mathbf{e} = \frac{1}{2}\left(\nabla \mathbf{u} + \nabla^T \mathbf{u}\right)$. To quantify how well linear elasticity theory (i.e., using the infinitesimal strain approximation) approximates finite elasticity theory, we define ξ to be the relative error between $\mathbf{E}$ and $\mathbf{e}$, i.e. $\xi = \|\mathbf{E} - \mathbf{e}\|_F / \|\mathbf{E}\|_F$ where $\|\bullet\|_F$ is the Frobenius norm of a tensor. The time variation of ξ at multiple locations around cell 1, cell 2, and cell 3 is shown in the upper left panels of Figs. S.5-S.7, with measurement locations shown in the other three panels of each figure. For these cells, we found that ξ is below 3% and gradually decreases over time, indicating that the cell-induced deformations in our experiment can be reasonably approximated by the infinitesimal strain approximation, and hence linear elasticity theory. In applying the MLS method, values of $d_m \approx 35$ μm and $r = 10^{-10}$ were used.

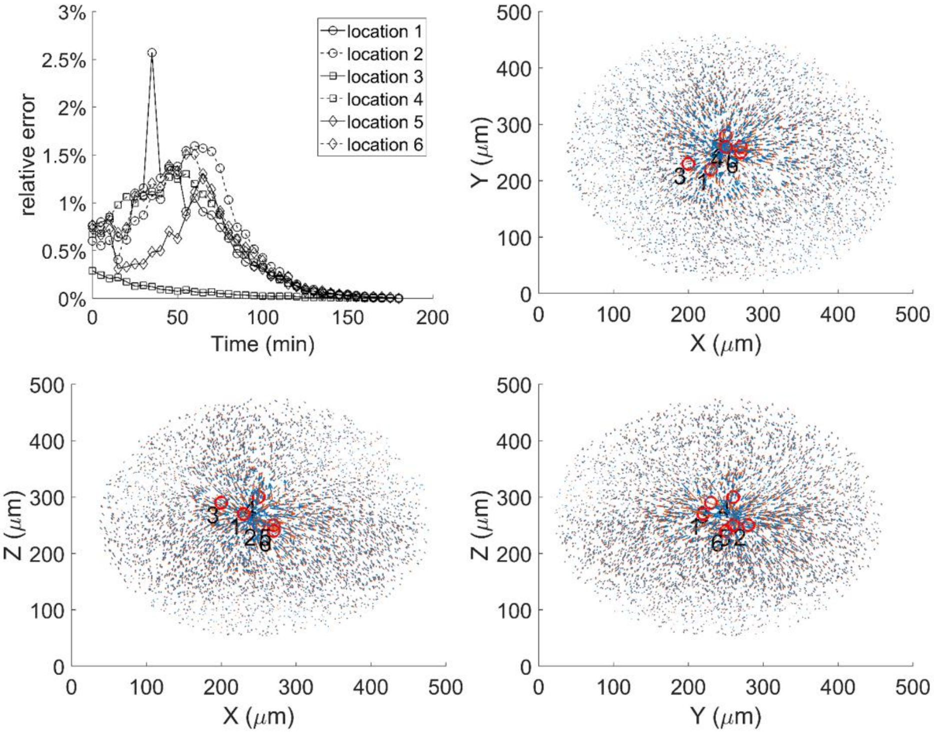

Figure S.5. Top left: the relative error between the finite strain tensor and infinitesimal strain tensor over time at multiple locations around cell 1. The specific locations are indicated in the top right (X-Y plane), bottom left (X-Z plane) and bottom right (Y-Z plane) panels in which the arrows indicate the cell-induced displacement.

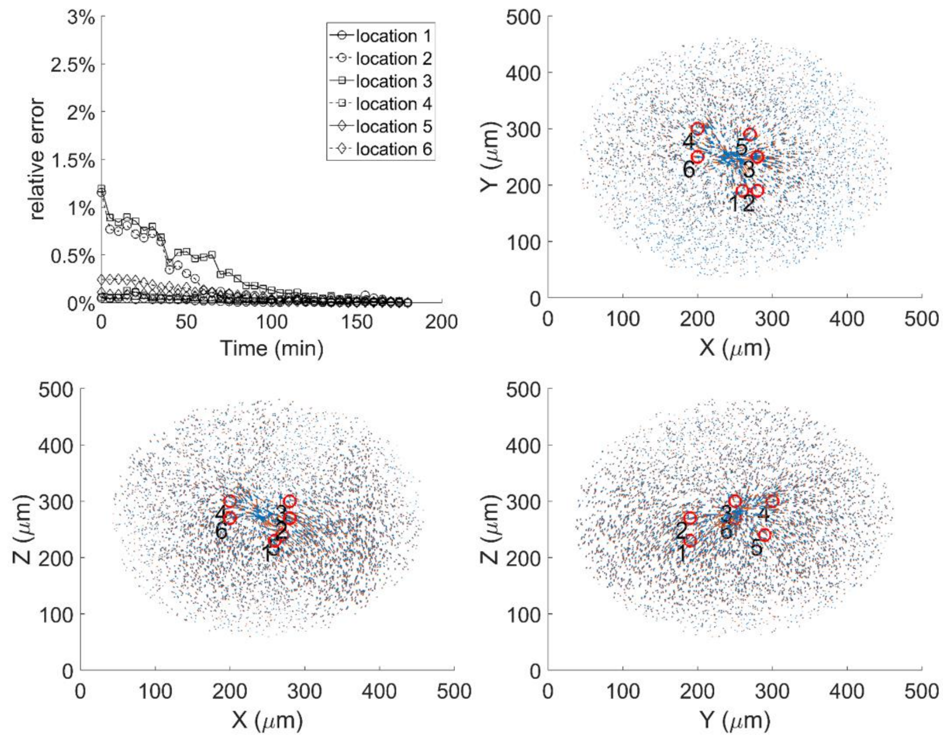

Figure S.6. Top left: the relative error between the finite strain tensor and infinitesimal strain tensor over time at multiple locations around cell 2. The specific locations are indicated in the top right (X-Y plane), bottom left (X-Z plane) and bottom right (Y-Z plane) panels in which the arrows indicate the cell-induced displacement.

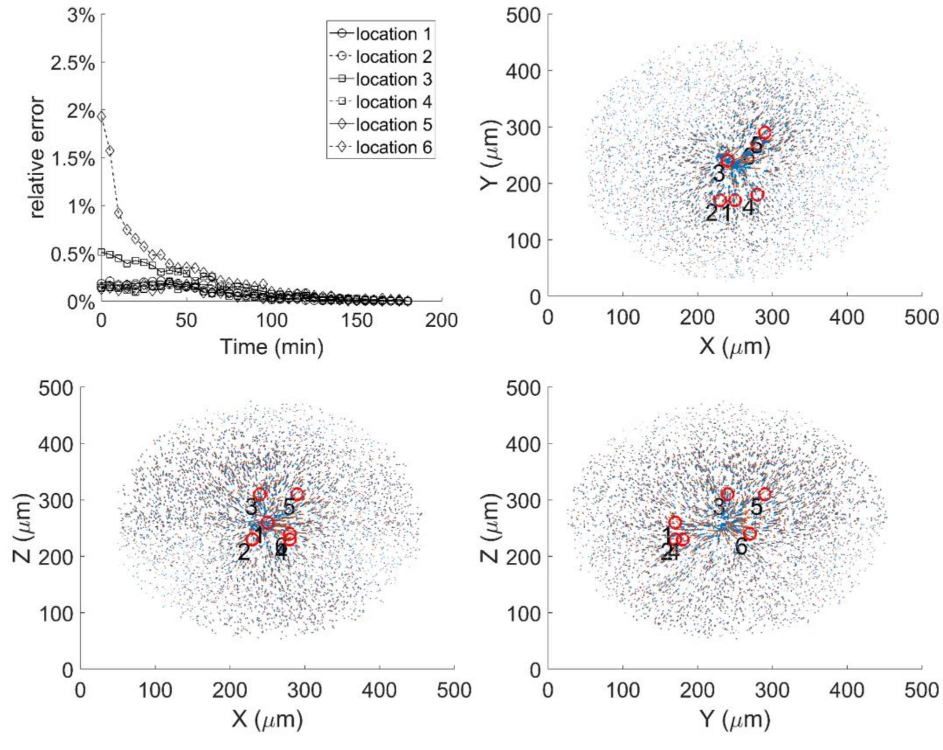

Figure S.7. Top left: the relative error between the finite strain tensor and infinitesimal strain tensor over time at multiple locations around cell 3. The specific locations are indicated in the top right (X-Y plane), bottom left (X-Z plane) and bottom right (Y-Z plane) panels in which the arrows indicate the cell-induced displacement.

Possible underestimation of CTFs due to residual stresses

Substrate deformations were obtained by relying on the assumption that the last time point in our time-lapse experiment corresponded to a 'proper' reference state, in which cell forces were completely relaxed. However, this assumption may fail due to the presence of residual stresses. Such stresses may either be active (i.e., due to incomplete cell relaxation) or passive (e.g., due to cell-induced substrate remodeling). TFM methods which generate a reference state via chemically-induced CTF inhibition cannot eliminate the possibility of residual passive stresses without additional *a priori* information about the sample structure prior to cellular activity (or alternatively, probing the substrate to detect changes in the local mechanical properties). Because the experimental protocol used for this study did not acquire any such data, our CTF reconstructions are vulnerable to inaccuracies resulting from the presence of passive stresses. Future TF-OCM protocols may be fortified against this vulnerability by acquiring a 'true' reference state immediately after sample preparation, which may act as a supplement or replacement for a chemically-induced reference state.

A loss of active stresses (in the case of nonlethal CTF inhibitors) may most readily be identified by the observation of a halt in mechanical activity. Upon closer inspection of our experimental data, we found reason to suspect that the cells shown in this study may not have completely relaxed by the reference time point (defined at t_{36}=3 hours). Specifically, we recomputed CTFs redefining both times t_{34} and t_{35} as reference points, and found that the total force curves (as in Fig. 8) displayed an average downward shift, indicating that the treated NIH-3T3 cells may not have completely relaxed by time t_{36} (not shown). Under a linear elastic model (the use of which was justified previously in this document), incomplete cell relaxation at the reference time point may have caused the CTF curves in Figs. 8 and 9 to be underestimated by an additive constant.

To estimate the constant factor by which CTFs may have been underestimated, we first extracted the 'force decay phase' of the curves in Fig. 9. The first time point in this phase was taken to be the time t_{peak} of the force peak after the application of cytochalsin D (i.e., any time after time t_6=30 minutes). All remaining time points (up to t_{36}) were included as part of the 'force decay phase' data. The extracted curves were fit to an exponential decay model of the form $F(t) = f_0 + f_1 e^{-t/\tau}$ via the MATLAB function *lsqcurvefit*. If t_{36} corresponded to a 'true' reference state, the coefficient f_0 would be expected to be zero. On the other hand, if residual active stresses were present during the reference state, f_0 would be expected to take a negative value. Using the value of f_0 obtained from the curve fit, we found that the total forces exerted by cells 1-10 may have been underestimated by approximately 6 nN, 56 nN, 4 nN, 1 nN, 28 nN, 11 nN, 5 nN, 1 nN, 0 nN, and 0 nN, respectively. As a percentage of the force measured at time $t = t_{\text{peak}}$ for each cell, these values correspond to an error of approximately 3%, 75%, 2%, 1%, 9%, 9%, 2%, 1%, 0%, and 0%, respectively. The degree of potential error for cells 2, 5, and 6 in particular, suggests that future experiments using cytochalasin D may require longer imaging times. This problem also highlights the need to develop real-time TF-OCM data processing so that researchers may reliably identify when it is appropriate to end a given time-lapse experiment. Future applications of TF-OCM to biological studies will require more thorough confirmation of effective CTF inhibition.

Factors reducing the sensitivity of CTF reconstructions

The sensitivity of TF-OCM to CTFs can be reduced by any effect which contributes noise or errors to the final reconstructed CTF data. The experiments detailed below were performed in order to better understand the impact of both localization sensitivity and bead density on the noise level of CTF reconstructions (and hence, the sensitivity of our TF-OCM system to CTFs). These experiments made use of artificially modified bead displacement data. That is, experimental data were altered (e.g., by adding noise or removing data points) to obtain multiple realizations of modified displacement data. By comparing the CTF reconstructions resulting from these modified data sets with the original CTF reconstructions, the impact of localization sensitivity and bead density could be studied in a controlled manner. It is important to note that the performance of our TF-OCM method emerges as a net consequence of the entire experimental system (including sample preparation, the imaging system, algorithms, etc.). As a consequence, these results are intended to demonstrate the limitations of only this first iteration of TF-OCM, and not the theoretical limitations of TF-OCM as a whole.

Impact of localization sensitivity

Modified bead displacement data were generated by adding Gaussian noise to experimental bead displacement data. The standard deviations of this Gaussian noise along the *x*, *y*, and *z* axes were given by the experimentally measured localization sensitivities defined in the Methods section of the main text (37 nm, 32 nm, and 86 nm, respectively). To prevent the addition of unrealistic/excessive noise, any noise realization with a magnitude greater than three standard deviations (along any axis) was discarded and replaced with another sample drawn from the Gaussian distribution. Ten independent realizations of this noise process were generated for each bead at each time point in the data sets of cells 1, 2, and 3 (yielding a total of 1110 modified bead displacement data sets). CTF reconstruction was performed using each of these data sets (requiring approximately 10 days of computing time on our work station). Total force curves (as in Figs. 8 and 9 in the main text) were generated from these reconstructions, and are displayed below in Fig. S.8.

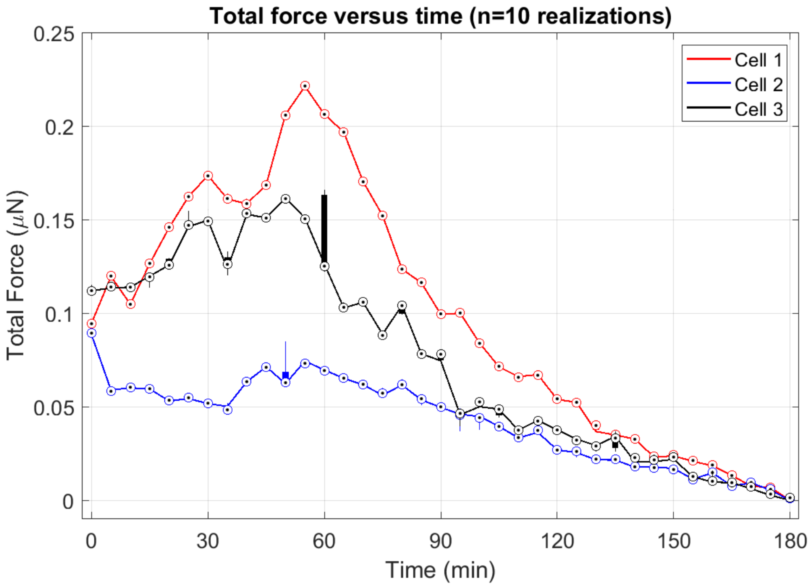

Figure S.8. Contribution of bead localization sensitivity to noise in reconstructed total force data. Original total force data (line plots, equivalent to data shown in Fig. 8) have been superimposed with total force data from the CTF reconstructions described in the text (box plots, obtained using bead displacement data modified with Gaussian noise). The whiskers, boxes, and circles depict the full range, interquartile range, and median value of the data, respectively. Note that most of the boxes exhibit so little deviation about the median data point that they effectively 'vanish' in the plot above.

The standard deviation of these new total force curves with respect to the original (unmodified) total force curves is 2.6 nN, which we interpret here as the approximate contribution of bead localization sensitivity to noise in the data shown in Figs. 8 and 9. These results suggest that the noise contributed by our bead localization sensitivity is so small as to be negligible in comparison to noise originating from other sources (such as low bead density, as detailed in the next section). It is important to note that, although the addition of Gaussian noise in this experiment had little impact on the measurement of total force, there are a few time points at which large deviations in total force can be seen. (e.g., such as cell 3 at time t=60 minutes). Such large deviations may result from multiple factors, such as failure of the FEM solver to converge and/or a low bead density contributing to numerical instability of the inverse problem posed by CTF reconstruction. This latter factor is explored by the experiment below.

Impact of bead density

Modified bead displacement data were generated via sub-sampling of experimental bead displacement data obtained from cell 1. Specifically, each bead was discarded from the data set with a probability of 50%, resulting in bead displacement data which had an *effective* bead density approximately half that of the original experimental data. This process was repeated to obtain 10 realizations of sub-sampled data for each time point. CTF reconstruction was performed using each of these data sets (requiring approximately 80 hours of computing time on our work station). Total force data (as in Figs. 8 and 9 in the main text) were generated from these reconstructions, and are displayed

below in Fig. S.9. We note that these results do not demonstrate the impact of bead density at the true bead density used in our samples. However, we assume that the features demonstrated by these ‘half-density’ data sets may serve as a sufficient proxy. Under the assumption that higher bead density reduces noise in CTF reconstructions, the noise measured using these ‘half-density’ datasets may serve as an approximate upper bound on the noise contribution of the true bead density present in the original experiment data.

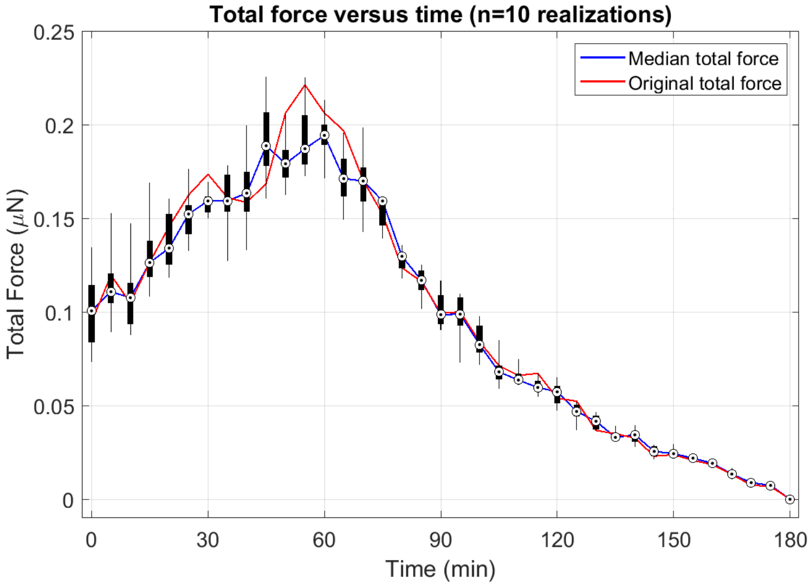

Figure S.9. Contribution of bead density to noise in reconstructed total force data (for cell 1). Box plots depict total force data from the CTF reconstructions described in the text (obtained using randomly sub-sampled realizations of the original bead displacement data). The whiskers, boxes, and circles depict the full range, interquartile range, and median value of the data, respectively. The median total force curve corresponds to the median values reported in the box plots. The original total force curve corresponds to the total force data from the CTF reconstructions obtained using the original experimental data.

There are several features worth discussing in these results. First, due to the relatively smooth contraction/relaxation of the cell (as in panel (b) of Supplementary Movie 1), we might have expected the original total force curve of cell 1 (the red curve in Fig. S.9) to vary smoothly across time. However, the curve appears ‘jagged’, possessing noisy/rapid fluctuations in the computed total force. We note that the peaks and troughs of this jagged curve are on the order of the variations in the total force obtained from the ‘half-density’ reconstructions shown in the box plots of Fig. S.9, which suggests that the fluctuations seen in the original cell 1 curve may be due to the impact of low bead density, and not ‘true’ fluctuations in CTFs. We also note that the curve traced by the median of the ‘half-density’ reconstructions appears relatively smooth, and therefore more closely resembles the type of time series that would have been expected, based on the image data. It must be emphasized that this information is not sufficient to determine whether this curve is closer to the ‘true’ cell force. However, if future experiments demonstrate such a finding, it could suggest that the effects of low bead density can be mitigated by adding stochastic elements (such as the random sub-sampling used here) to force reconstruction procedures.

The variation of total force in Fig. S.9 is not uniform over time. In particular, the standard deviation of the total force obtained from the ‘half-density’ reconstructions increases with increasing time between the ‘deformed state’ and ‘reference state’ (shown in Fig. S.10). If we consider this time-varying standard deviation to represent the noise in our TF-OCM system, we see that noise increased at a rate of approximately 0.1 nN/min of temporal separation between the ‘deformed’ and ‘reference’ states. This implies that our TF-OCM system exhibited its worst sensitivity at time t=0, with a sensitivity/noise floor of approximately 18 nN (which is approximately one tenth the median cell force measured at time t=0, as shown in Fig. 9). Note that this noise floor approximates the contribution of our (relatively) low bead density. As stated in the Discussion, our 1 μm diameter beads had an average separation of 18 μm, but standard TFM conventions would allow for a separation as small as 10 μm, corresponding to a bead density almost 6 times larger than that used in this study. It is currently unknown whether the noise floor calculated/inferred from the ‘half-density’ CTF reconstructions shown here captures the majority of the noise observed in our experimental measurements of total force. Future experiments using higher bead densities will be likely to add clarity to this

question. The interpretation of such experiments will also require further analysis of the factors detailed in the paragraphs below. At minimum, the noise measured in this experiment may be considered as an approximate lower bound on the reconstruction noise in our TF-OCM system (e.g., at the time of 'worst performance', time t=0, our method could not reliably measure total forces smaller than approximately 20 nN).

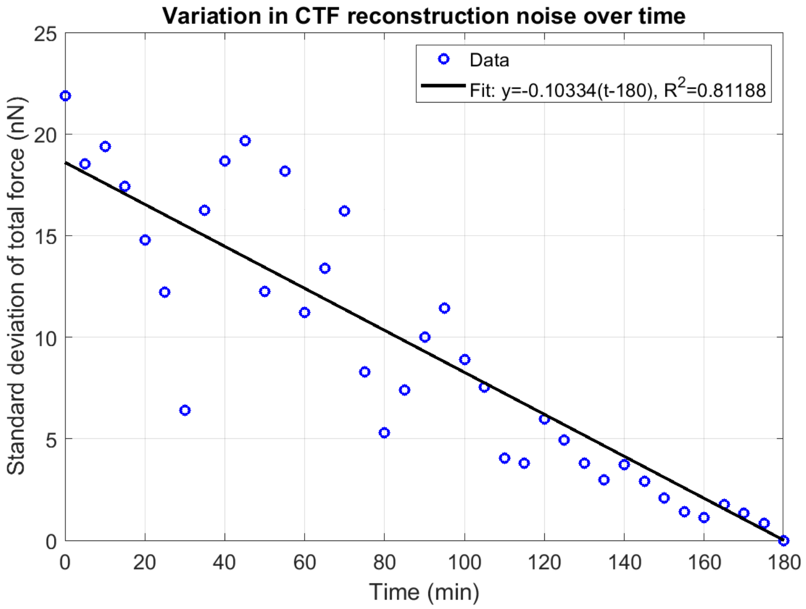

Figure S.10. Standard deviation of the total force data shown in the box plots of Fig. S.9. These results appear to indicate that the sensitivity of our TF-OCM method to CTFs decreases as the time between the 'deformed' and 'reference' states increases. See text for details.

There are multiple possible explanations for a time-varying sensitivity to CTFs. The fact that the noise floor changes approximately linearly in time *despite* the introduction of a contractility inhibitor at time t=30 minutes suggests that strain/deformation dependent effects are unlikely to be the primary culprit. If such effects were at fault, we would expect the data in Fig. S.10 to show a change in behavior before versus after the addition of cytochalasin D. A reliable test of this hypothesis would likely require experiments with an extended observation time prior to the addition of cytochalasin D, beyond the 30 minutes used in this study.

Imaging and data processing effects may be more likely causes of time-varying sensitivity to CTFs. Errors in bead tracking may increase with increasing time from the 'reference' time point. Alternatively (or in addition), sample drift may play a significant role. Although bead displacement data were corrected for bulk (zero-order) and linear (first-order) drift, a sufficiently large drift of the sample may cause the assumptions underlying our computational image formation module to fail, inducing higher-order image deformations which our algorithms do not currently accommodate. Coherence gate curvature correction and focal plane registration are likely candidates to suffer from such an effect. The surprisingly small standard deviation in the 'half-density' reconstruction in the latter half of the time-lapse suggest that such time-varying image distortions may be of greater concern than bead density. (That is, although a low bead density is not desirable, its impact may be amplified by the presence of image distortions.) Therefore, these results suggest that future iterations of TF-OCM may benefit from further exploration and refinement of our computational image formation procedures.

Supplementary References

1 Graf, B. W., Adie, S. G. & Boppart, S. A. Correction of coherence gate curvature in high numerical aperture optical coherence imaging. *Opt Lett* **35**, 3120-3122, doi:10.1364/OL.35.003120 (2010).

2 Adie, S. G., Graf, B. W., Ahmad, A., Carney, P. S. & Boppart, S. A. Computational adaptive optics for broadband optical interferometric tomography of biological tissue. *Proc Natl Acad Sci U S A* **109**, 7175-7180, doi:10.1073/pnas.1121193109 (2012).

3 Kumar, A. *et al.* Anisotropic aberration correction using region of interest based digital adaptive optics in Fourier domain OCT. *Biomedical Optics Express* **6**, 1124-1134, doi:10.1364/Boe.6.001124 (2015).

4 Ralston, T. S., Marks, D. L., Carney, P. S. & Boppart, S. A. Interferometric synthetic aperture microscopy. *Nat Phys* **3**, 129-134, doi:10.1038/nphys514 (2007).

5 Shemonski, N. D. *et al.* Stability in computed optical interferometric tomography (part I): stability requirements. *Opt Express* **22**, 19183-19197, doi:10.1364/OE.22.019183 (2014).

6 Shemonski, N. D. *et al.* Stability in computed optical interferometric tomography (part II): in vivo stability assessment. *Opt Express* **22**, 19314-19326, doi:10.1364/OE.22.019314 (2014).

7 Liu, S., Lamont, M. R. E., Mulligan, J. A. & Adie, S. G. Aberration-diverse optical coherence tomography for suppression of multiple scattering and speckle. *Biomed Opt Express* **9**, 4919-4935 (2018).

8 Mulligan, J. A., Bordeleau, F., Reinhart-King, C. A. & Adie, S. G. in *Biomechanics in Oncology* (eds C. Dong, N. Kuhn, & K. Konstantopoulos) (Springer Nature Switzerland AG, In Press, 2018).

9 Feng, X. Z. & Hui, C. Y. Force sensing using 3D displacement measurements in linear elastic bodies. *Comput Mech* **58**, 91-105, doi:10.1007/s00466-016-1283-1 (2016).

10 Nam, S., Hu, K. H., Butte, M. J. & Chaudhuri, O. Strain-enhanced stress relaxation impacts nonlinear elasticity in collagen gels. *Proc Natl Acad Sci U S A* **113**, 5492-5497, doi:10.1073/pnas.1523906113 (2016).

11 Toyjanova, J. *et al.* 3D Viscoelastic traction force microscopy. *Soft Matter* **10**, 8095-8106, doi:10.1039/c4sm01271b (2014).

12 Hall, M. S., Long, R., Hui, C. Y. & Wu, M. M. Mapping Three-Dimensional Stress and Strain Fields within a Soft Hydrogel Using a Fluorescence Microscope. *Biophys J* **102**, 2241-2250, doi:10.1016/j.bpj.2012.04.014 (2012).
